# Supplementary material for: Prediction Model for Hypertension and Diabetes Mellitus Using Korean Public Health Examination Data (2002–2017)
Source: Diagnostics (Basel). 2022 Aug 14;12(8):1967. doi: 10.3390/diagnostics12081967 (PMC9407141; doi:10.3390/diagnostics12081967)
Supplement: Supplementary file 1 [file diagnostics-12-01967-s001.zip › diagnostics-1828030-supplementary.pdf]

diagnostics

MDPI

Table S1. Pharmaceutical ingredient codes for hypertension medication.

111001ACE 216604ATB 502201ATB 526900ATB 111001ATB 227801ATB 502202ATB 527000ATB 111001ATE 227801ATR 502203ATB 527100ATB 111002ATE 227802ATB 502204ATB 614500ATB 111003ACE 227803ATB 507800ATB 629700ATB 111003ATE 227805ATB 517900ACH 629800ATB 111501ATB 454001ATB 518900ATB 629900ATB 111502ATB 454002ATB 519300ACH 630000ATB 111503ATB 454003ATB 524000ATB 630100ATB 111504ATB 470901ATB 524100ATB 630200ATB 162401ACH 470902ATB 525000ATB 631400ATB 162402ACH 470903ATB 525100ATB 631500ATB 162403ATR 471000ATB 525200ATB 631600ATB 185801ATB 471100ATB 525300ATB 631700ATB 216601ATB 472300ATB 526300ATB 216602ATB 472400ATB 526400ATB 216603ATB 472500ATB 526500ATB

Table S2. Pharmaceutical ingredient codes for diabetes medication.

100601ATB 100602ATB 165402ATB 165602ATB 165603ATR 165604ATR 165701ATB 165702ATB 165703ATB 165704ATB 165801ATB 191501ATB 191502ATB 191502ATR 191503ATB 191504ATB 191504ATR 191505ATR 249001ATB 249001ATD 249002ATB 249002ATD 379501ATB 379502ATB 379503ATB 406201ATB 430201ATB 430202ATB 430203ATB 431901ATB 431902ATB 443400ATB 443500ATB 471900ATB 474200ATB 474300ATB 474300ATR 486101ATB 497200ATB 498100ATB 498600ATB 500801ATB 501101ATB 501102ATB 501103ATB 502300ATB 502300ATR 502900ATB 507000ATB 507100ATB 513700ATB 513700ATR 518500ATR 518600ATR 518800ATB 519600ATB 520500ATB 520600ATB 520700ATB 523600ATB 523700ATB 523800ATR 524700ATR 525500ATB 525600ATB 525901ATB 527301ATB 527302ATB 613301ATB 613302ATB 616401ATB 619101ATB 624201ATB 624202ATB 624203ATB 627301ATB 628201ATB 628202ATB 630300ATB 630400ATB 630500ATB 630600ATB 631900ATB 632000ATR 632100ATB 635600ATB 635700ATB 636101ATB 637200ATB 639601ATB 639800ATR 641400ATR 641800ATR 641900ATR 642000ATR 644900ATB 645000ATR 645301ATB 648400ATB 648500ATB 648600ATB 649000ATB 649100ATB 649200ATB 649300ATB 649400ATB 649500ATB 649900ATR 650000ATR 650100ATR 653800ATR 653900ATR 654000ATR 654100ATR 655700ATR 664600ATB 664700ATB 664800ATB 170130BIJ 170131BIJ 170430BIJ 170431BIJ 175330BIJ 175331BIJ 175332BIJ 175333BIJ 441330BIJ 441331BIJ 441332BIJ 441333BIJ 441334BIJ 461830BIJ 461831BIJ 461832BIJ 484930BIJ 484931BIJ 488730BIJ 512130BIJ 512131BIJ 626631BIJ 626630BIJ 626700BIJ 626830BIJ 626831BIJ 639701BIJ 639702BIJ 644501BIJ 644502BIJ

Table S3. Baseline characteristics after Propensity Score Matching (Addition of family history variables of diabetes and hypertension.

Variables	Total (n=480,290)	Screening < 5 times (N = 240,145)	Screening ≥ 5 times (N =240,145)	*P* value
**Sex**				0.9804
male	321,822(67.01)	160,907(67.00)	160,915(67.01)	
female	158,468(32.99)	79,238(33.00)	79,230(32.99)	
**Age, years**	52.88(11.43)	52.88(11.43)	52.87(11.42)	0.8568
				0.9992
30s	64,293(13.39)	32,146(13.39)	32,147(13.39)	
40s	122,950(25.60)	61,497(25.61)	61,453(25.59)	
50s	156,431(32.57)	78,173(32.55)	78,258(32.59)	
60s	92,883(19.34)	46,449(19.34)	46,434(19.34)	
70s	43,733(9.11)	21,880(9.11)	21,853(9.1)	
**BMI, kg/m^2**				<0.0001
< 18.5	7,651(1.59)	4,230(1.76)	3,421(1.42)	
18.5 – 22.9	133,726(27.84)	66,596(27.73)	67,130(27.95)	
23.0 – 24.9	126,171(26.27)	61,440(25.58)	64,731(26.95)	
≥ 25.0	212,742(44.29)	107,879(44.92)	104,863(43.67)	

**Diastolic blood pressure, mmHg**	80.72(10.45)	80.65(10.56)	80.78(10.33)	0.0302
				0.6490
≤ 79	178,739(37.21)	89,557(37.29)	89,182(37.14)	
80 - 89	206,802(43.06)	103,195(42.97)	103,607(43.14)	
90 - 99	62,908(13.10)	31,455(13.10)	31,453(13.10)	
≥ 100	31,841(6.63)	15,938(6.64)	15,903(6.62)	
**Systolic blood pressure, mmHg**	125.97(15.14)	128.90(15.35)	129.00(14.94)	0.0437
				0.5519
≤ 119	109,865(22.87)	55,133(22.96)	54,732(22.79)	
120 - 139	266,249(55.44)	132,923(55.35)	133,326(55.52)	
140 - 159	85,124(17.72)	42,573(17.73)	42,551(17.72)	
≥ 160	19,052(3.97)	9,516(3.96)	9,536(3.97)	
**Fasting blood sugar, mg/dL**	98.34(21.75)	98.46(23.42)	98.21(19.95)	0.0628
				0.9967
< 100	311,192(64.79)	155,598(64.79)	155,594(64.79)	
100 -126	145,744(30.34)	72,876(30.35)	72,868(30.34)	
> 126	23,354(4.86)	11,671(4.86)	11,683(4.86)	
**Total cholesterol, mg/dL**	200.34(38.81)	200.90(40.59)	199.80(36.93)	<0.0001
**Alcohol consumption, times/week**				<0.0001
0	227,618(47.39)	116,231(48.40)	111,387(46.38)	
1	89,183(18.57)	39,925(16.63)	49,258(20.51)	
2,3	115,146(23.97)	54,458(22.68)	60,688(25.27)	
4-7	48,343(10.07)	29,531(12.30)	18,812(7.83)	
**Smoking**				<0.0001
never	251,124(52.29)	125,514(52.27)	125,610(52.31)	
ex	95,805(19.95)	41,025(17.08)	54,780(22.81)	
current	133,361(27.77)	73,606(30.65)	59,755(24.88)	
**Physical activity, times/week**	910.91(1,200.73)	703.80(1,126.90)	1,1180.00(1,236.10)	<0.0001
**Outcomes**				
**Hypertension**				<0.0001
no	353,297(73.56)	166,457(69.32)	186,840(77.80)	
yes	126,993(26.44)	73,688(30.68)	53,305(22.20)	
**Diabetes mellitus**				<0.0001
no	291,700(60.73)	138,978(57.87)	152,722(63.60)	
yes	188,590(39.27)	101,167(42.13)	87,423(36.40)	
**Hypertension and diabetes mellitus**				<0.0001
no	437,450(91.08)	213,088(88.73)	224,362(93.43)	
yes	42,840(8.92)	27,057(11.27)	15,783(6.57)	

Data are presented as a n (%) or mean (SD).

Table S4. Logistic regression of hypertension and diabetes mellitus according to screening frequency.

	Hypertension	Diabetes Mellitus	Hypertension and Diabetes Mellitus
Variable	OR (95% CI) *	OR (95% CI) **	OR (95% CI) ***
Screening frequency			
1	2.18(2.13-2.23)	1.39(1.36-1.41)	2.49(2.41-2.57)
2	1.53(1.49-1.57)	1.22(1.19-1.24)	1.71(1.65-1.77)

3	1.25(1.22-1.28)	1.10(1.08-1.13)	1.34(1.29-1.39)
4	1.10(1.07-1.12)	1.07(1.05-1.10)	1.18(1.13-1.22)
5	Ref.	Ref.	Ref.
6	1.04(1.01-1.07)	0.94(0.92-0.97)	1.03(0.98-1.08)
7	1.04(1.01-1.08)	0.92(0.89-0.95)	1.02(0.97-1.08)
8	0.98(0.94-1.01)	0.90(0.87-0.93)	0.96(0.90-1.01)
9	0.91(0.88-0.95)	0.87(0.84-0.90)	0.90(0.85-0.96)
10	0.83(0.80-0.85)	0.83(0.80-0.85)	0.80(0.76-0.85)

* Adjusted by age, sex and family history of hypertension
** Adjusted by age, sex and family history of Diabetes Mellitus
*** Adjusted by age, sex, family history of hypertension and Diabetes Mellitus

Table S5. Logistic regression of hypertension and diabetes mellitus according to screening frequency.

	**Hypertension**		**Diabetes Mellitus**		**Hypertension and Diabetes Mellitus**	
**Variables**	**OR (95% CI)**	**Adjusted OR* (95% CI)**	**OR (95% CI)**	**Adjusted OR** (95% CI)**	**OR (95% CI)**	**Adjusted OR*** (95% CI)**
**Screening frequency**						
≥5 times	Ref.	Ref.	Ref.	Ref.	Ref.	Ref.
<5 times	1.55(1.53-1.57)	1.58 (1.55-1.60)	1.27(1.26-1.29)	1.28 (1.26-1.29)	1.81(1.77-1.84)	1.80 (1.76-1.84)
**Age**						
30s	Ref.	Ref.	Ref.	Ref.	Ref.	Ref.
40s	0.72(0.71-0.73)	0.72(0.71-0.74)	1.30(1.28-1.33)	1.31(1.28-1.33)	0.87(0.85-0.90)	0.88(0.86-0.91)
50s	0.45(0.44-0.46)	0.48(0.47-0.49)	1.75(1.72-1.79)	1.76(1.72-1.79)	0.67(0.65-0.69)	0.73(0.71-0.75)
60s	0.30(0.30-0.31)	0.33(0.32-0.33)	2.33(2.28-2.38)	2.35(0.30-2.40)	0.52(0.50-0.54)	0.57(0.55-0.59)
70s	0.22(0.21-0.22)	0.23(0.22-0.23)	2.76(2.69-2.83)	2.79(2.72-2.87)	0.40(0.38-0.42)	0.44(0.42-0.46)
**Sex**						
male	Ref.	Ref.	Ref.	Ref.	Ref.	Ref.
female	0.47(0.46-0.48)	0.56(0.55-0.57)	1.18(1.16-1.19)	1.03(1.02-1.05)	0.55(0.53-0.56)	0.61(0.59-0.62)
**Family history of**						

**hypertension**						
no	Ref.	Ref.	-	-	Ref.	Ref.
yes	0.62(0.61-0.63)	0.58(0.57-0.59)	-	-	0.60(0.59-0.62)	0.58(0.56-0.60)
**Family history of Diabetes Mellitus**						
no	-	-	Ref.	Ref.	Ref.	Ref.
yes	-	-	1.04(1.02-1.06)	1.17(1.15-1.20)	1.19(1.16-1.23)	1.27(1.23-1.31)
**AUC**	-	0.67	-	0.59	-	0.64

* Adjusted by age, sex and family history of hypertension
** Adjusted by age, sex and family history of Diabetes Mellitus
*** Adjusted by age, sex, family history of hypertension and Diabetes Mellitus
OR, Odds Ratio; CI, Confidence Interval; Ref., reference.

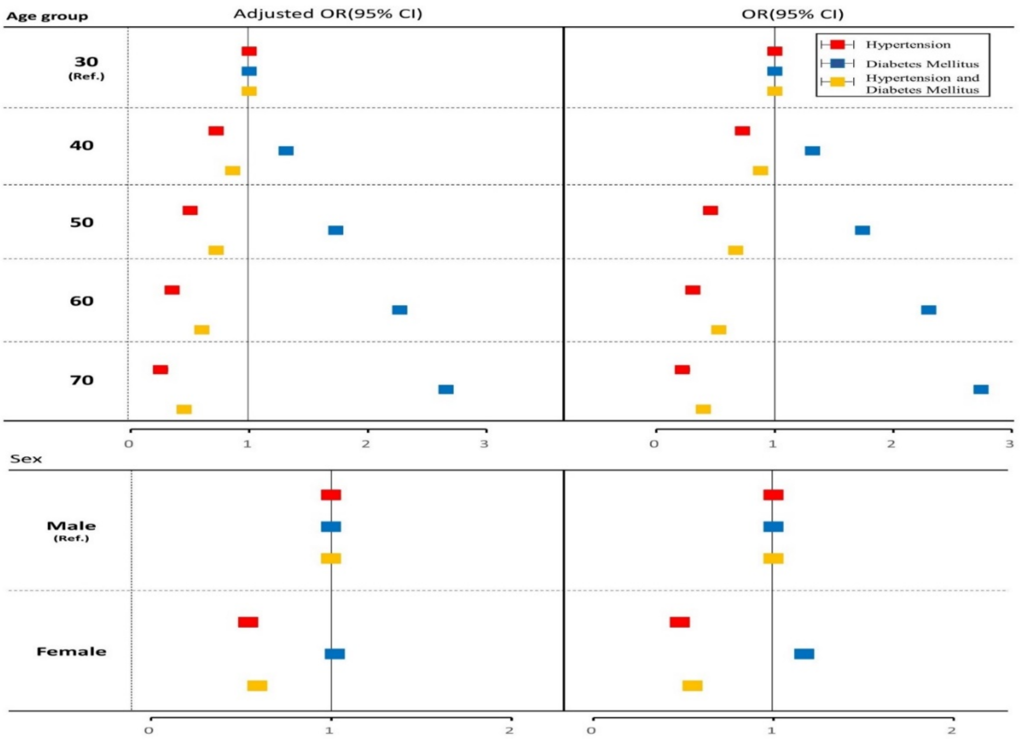

Figure S1. Sensitivity analysis results
